# Who’s to blame for central venous stenosis

**DOI:** 10.12669/pjms.39.4.7559

**Published:** 2023

**Authors:** Xiaodong Li, Shuangyan Liu

**Affiliations:** 1 Xiaodong Li, Department of Nephrology, Baoding No. 1 Central Hospital of Hebei Medical University, Baoding, Hebei, China; 2 Shuangyan Liu, Graduate School of Hebei Medical University, Shijiazhuang 050017, Hebei, China

Chronic kidney disease (CKD) has become a worldwide public health problem and its prevalence is about 13.9%.^1^ There are about 120 million CKD patients in China, around 2% of whom will go into end-stage renal disease (ESRD) and require dialysis or kidney transplantation. Furthermore, with the rising of aging, diabetes and hypertension, the number of ESRD patients keeps increasing. The ESRD patients receiving hemodialysis normally need to be established vascular access first. Although autogenous arteriovenous fistula is preferred for vascular access for hemodialysis, more and more ESRD patients choose to be placed with central venous catheter (CVC) due to their own poor vascular conditions and short life expectancy.^2^ There are many complications of application in CVC, such as infection, thrombosis, embolism and central vascular stenosis (CVS). As the lifetime of hemodialysis patients becomes longer, CVS is becoming a major obstacle to threaten the lives of those patients with CVC.^3^

There are no exact data on the incidence of CVS due to the asymptomatic manifestations of those patients in the early stage. However, as the disease progresses, CVS may lead to multiple clinical symptoms and even rare complications, such as superior vena cava syndrome (SVCS), which poses a tremendous threat to CVS patients with hemodialysis.^3^ There are many factors influencing CVS, including vascular device placement, anatomical factors, and hemodynamic changes. Among them, the most important one is central vascular catheterization.^4^ The incidence of CVS in hemodialysis patients with long-term CVC is nearly 20% to 40%.^3^ The pathogenesis of CVS has not been clearly clarified to date, which may be related to the vascular endothelial damage leading to the production of inflammatory response and the accumulation of thrombosis, collagen and endothelial cells.^3^

Hemodialysis patients with CVS generally show swelling of the arm, head, neck, or trunk, which may be unilateral or bilateral. In addition, CVS may cause neurological symptoms, pleural effusion, dyspnea and even SVCS. Most hemodialysis patients are usually asymptomatic because their degree of CVS is lower 50%. The Guidelines from kidney disease outcomes quality initiative (KDOQI) do not recommend early intervention in asymptomatic CVS, but it may be selected for therapy based on imaging techniques.^5^ Digital subtraction angiography is the golden standard imaging diagnostic method but invasive, while color-doppler ultrasound, computed tomography angiography, and magnetic resonance angiography are non-invasive imaging and still controversial for central venous imaging. It is expected that there will be non-invasive imaging approach and more imaging models for CVS in the future.

Endovascular treatment is recommended as the first selection for symptomatic CVS, which can maximize the utilization of autogenous vascular resources.^5^ However, repeated endovascular interventions are required for forward patency. If there exists repeated endovascular interventions for CVS and central venous occlusion, surgical reconstruction of vascular access is necessary, but it is an open surgery with vast trauma and high risk, and its long-term patency rate is not clear.^4^ It is advocated that the multiple collaboration of experts from endovascular intervention, vascular surgery, radiology and nephrology improve and perfect the recanalization technology for the treatment for CVS. In conclusion, how to avoid central CVS? The optimal reply may be to avoid using CVC.

## Authors’ contributions:

**XL:** Design, writing and revision. **SL:** Literature review. All authors contributed to the article and approved the submitted version.
